# Predicting short-term weight loss using four leading health behavior change theories

**DOI:** 10.1186/1479-5868-4-14

**Published:** 2007-04-20

**Authors:** António L Palmeira, Pedro J Teixeira, Teresa L Branco, Sandra S Martins, Cláudia S Minderico, José T Barata, Sidónio O Serpa, Luís B Sardinha

**Affiliations:** 1 Faculty of Human Movement, Technical University of Lisbon, Estrada da Costa, 1495-688, Cruz-Quebrada, Portugal; 2 University Lusófona de Humanidades e Tecnologias, Campo Grande, 1749-028, Lisbon, Portugal

## Abstract

**Background:**

This study was conceived to analyze how exercise and weight management psychosocial variables, derived from several health behavior change theories, predict weight change in a short-term intervention. The theories under analysis were the Social Cognitive Theory, the Transtheoretical Model, the Theory of Planned Behavior, and Self-Determination Theory.

**Methods:**

Subjects were 142 overweight and obese women (BMI = 30.2 ± 3.7 kg/m^2^; age = 38.3 ± 5.8y), participating in a 16-week University-based weight control program. Body weight and a comprehensive psychometric battery were assessed at baseline and at program's end.

**Results:**

Weight decreased significantly (-3.6 ± 3.4%, p < .001) but with great individual variability. Both exercise and weight management psychosocial variables improved during the intervention, with exercise-related variables showing the greatest effect sizes. Weight change was significantly predicted by each of the models under analysis, particularly those including self-efficacy. Bivariate and multivariate analyses results showed that change in variables related to weight management had a stronger predictive power than exercise-specific predictors and that change in weight management self-efficacy was the strongest individual correlate (p < .05). Among exercise predictors, with the exception of self-efficacy, importance/effort and intrinsic motivation towards exercise were the stronger predictors of weight reduction (p < .05).

**Conclusion:**

The present models were able to predict 20–30% of variance in short-term weight loss and changes in weight management self-efficacy accounted for a large share of the predictive power. As expected from previous studies, exercise variables were only moderately associated with short-term outcomes; they are expected to play a larger explanatory role in longer-term results.

## Background

Obesity and excessive weight are common concerns among people in industrialized countries. Scientific literature consistently reports the epidemic status of obesity [e.g., [1-3]], however, the progress that has been made in the study of biological, psychosocial and environmental processes related to weight management is still far from offering the desired integrated solutions. One of the greatest quests in this area is, therefore, to congregate these findings into comprehensive treatment programs that can counter the present situation [4].

Albeit reported inconsistently in the literature, psychosocial variables are accepted as playing a key role in explaining weight management [4,5]. These variables are commonly gathered in health behavior models such as the Theory of Planned Behavior [TPB – [6]], the Transtheoretical Model [TTM – [7]], or more comprehensive human behavior theories like the Social-Cognitive Theory [SCT – [8]] and Self-Determination Theory [SDT – [9]].

The SCT is the most frequently used paradigm in weight management interventions [10] and it is also commonly used to design physical activity interventions [e.g., [11,12]]. This theory is based on the reciprocal determinism between behavior, environment, and person, with their constant interactions constituting the basis for human action [13]. In this scenario, self-efficacy beliefs operate concurrently with cognized goals, outcome expectations, and perceived barriers and facilitators as fundamental constructs in the understanding of human agency, including health behaviors [14]. Agency is therefore a function of the degree a person believes she/he can complete the specific action. The construct of self-efficacy has been among the most analyzed psychosocial constructs in both nutrition [15,16] and physical activity studies [e.g., [17,18]]. It represents the most powerful determinant within SCT [10] although it is often not complemented by other SCT constructs in comprehensive predictive models [e.g., [19]]. Perceived barriers and expected outcomes are other SCT constructs that have been used before in weight control studies [e.g., [20,21]].

The TPB suggests that a person's behavior is determined by intentions to engage in that behavior and by one's perceived behavior control (PBC). Intentions sustain the motivational factors that influence the behavior, reflecting how much effort the person is willing to exert to perform the behavior. PBC is the degree of confidence perceived by the person regarding her/his ability to perform the behavior, and it is influenced by the beliefs towards resources and opportunities. Intentions are determined by PBC, attitudes, and subjective norms, where attitudes are the evaluation and beliefs towards the result of the behavior, and subjective norms the perceived pressure from significant others for the completion of the behavior [6]. In a meta-analytical study, the TPB has been shown to predict about 20% of actual exercise and nutrition behaviors [22]. The adoption of health behaviors is expected to increase substantially when specific plans to take goals into practice (named *implementation intentions*) are also part of the behavior change intervention [23]. Requiring participants to explicitly specify *when, where, how *they will engage in particular behaviors, that is, inducing change from a motivational to a volitional phase of behavior regulation, has been shown to increase the predictive ability of the TPB regarding exercise [24]. Relevant to the topic of the present study, the TPB has been used to explain several eating-related [e.g., [25,26]] and exercise behaviors [e.g., [27,28]], with similar results to those reported in the meta-analytical study.

The TTM uses several constructs from other health behavior theories, in a model that offers a view of when, how, and why people change their behavior [7]. This model includes two levels: i) the *stages of change *(SOC), which reflect the temporal dimension of the behavior, divided in six consecutive stages; and ii) a set of constructs that explain how people evolve along the SOC. These are named *processes of change*, i.e. cognitive and behavioral activities that individuals use to modify their experiences and environments to obtain the desired behavior. Also included in this model are the *decisional balance*, representing the pros and cons of engaging in the behavior, and *self-efficacy*, reflecting the person's confidence in performing the health behavior change [7]. The TTM has been extensively used both in nutrition [e.g., [29]] and exercise settings [e.g., [12], e.g., [30]], mainly because of its practical use in building stage-tailored interventions. The TTM has gathered support mainly on the exercise setting, although methodological problems have restrained meta-analytical studies to put forward a clear conclusion about the effectiveness of the theory to predict behavior [30]. Studies on nutrition also present some methodological problems, leading to inconclusive findings about the effectiveness of the TTM [10]. Weight management interventions with the TTM that have targeted both nutrition and physical activity behaviors are less common. In one study, Jeffery and colleagues showed that the SOC at baseline were not associated with weight loss over a three-year period in a large sample, although a tendency toward greater weight loss was present in the more active SOC [31]. This study only evaluated the SOC level of the TTM and did not account for past weight loss experiences, which could have contributed to the small predictive power of the variables presented by the TTM as a whole [31]. Suris and colleagues also built a weight loss intervention with 81 American-Mexican women based on the TTM [32]. In this study, the original staging algorithm was changed to reflect the particular patterns of obesity treatment practices observed among the participants. These results suggest that there may be culture-based biases on the evolution of the processes of change as predicted in the TTM original design.

The SDT is a motivation theory that highlights people's inherent need to evolve and to be integrated in a social scenario. Three primary needs that have been identified are competence, relatedness, and autonomy, which lead to different types of motivation to act, the most important and desirable being intrinsic motivation. This construct reflects our inherent tendency to seek out novelty and challenge, while feeling competent and autonomous in the process. Enjoyment, mastery, and positive feelings arise from this quest, reinforcing the continuation of the behavior. In opposition, extrinsic motivation is more externally driven, more controlled (i.e., less autonomous), and more disconnected from the behavior itself (more focused on its outcomes). Lastly, amotivation is a state where there is a lack of intention to act so that the outcome behavior has no personal value and feelings of competence are not present [9]. The SDT has been used in nutrition [e.g., [33]], exercise [e.g., [34]], and weight management settings [e.g., [35,36]] with positive results. In a recent study, increases in exercise self-efficacy and reductions in exercise perceived barriers were correlated with short-term weight loss, while only change in exercise intrinsic motivation was an independent predictor of long-term weight loss [35].

The previous theories constitute science' best effort to explain how peoples' decisions and choices toward exercise and healthy nutrition are built [37]. They are generally motivation-oriented, i.e., representing behavior as a proxy effect of the increase or high values on motivation. In the present study, the focus is primarily on the formation of motivation, attempting to fill a gap in the literature, where only a reduced number of studies have analyzed the predictive power of multiple psychosocial variables and different models [e.g., [27]]. Questions remains about which model or set of variables could better explain the outcomes of choice, which constructs may overlap, or if a set of variables from different theories could delineate the way to a new paradigm. Rothmam [38] highlights this last aspect as a likely cause of some of the disappointing results for most studies of behavior change interventions conducted to date.

Building on recent discussions on the usefulness of theory-based interventions in health behavior promotion [38-40] and following our analysis of baseline predictors of weight loss [41], the purpose of this study was to investigate the predictive value of changes in exercise and weight management related variables on weight change, in a sample of overweight and moderately obese women participating in a University-based weight management program. The constructs analyzed were selected as representative of the Social-Cognitive Theory, the Transtheoretical Model, the Theory of Planned Behavior, and Self-Determination Theory.

## Methods

### Participants

Participants were recruited from the community for a 2-year weight management program through newspaper ads, a website, email messages on listservs, and announcement flyers. Subjects were required to be older than 24 years, be premenopausal and not currently pregnant, have a BMI higher than 24.9 kg/m^2^, and be free from major disease to be eligible for the study. After the selection process 142 overweight and obese women (BMI = 30.2 ± 3.7 kg/m^2^; Age = 38.3 ± 5.8 y) started the program. For this study, only the first four months are being analyzed, a period during which all participants received the same intervention; they were later randomized to two different long-term programs or to controls. Attrition was 6.3% from baseline to 4 months (133 completers). However, some psychometric data were incomplete due to errors in the completion of some questionnaires either on baseline or after the intervention, leading to smaller sample sizes in some analyses.

### Intervention

The intervention was composed of fifteen weekly meetings, which lasted 120 minutes, and where both educational and practical components were scheduled. Attendance averaged 83% and groups were composed of 32–35 women, who entered the study in two cohorts. The intervention has been described before [41] and was loosely based on the LEARN weight management program [42], which generally follows a social-cognitive approach. Aspects such as self-efficacy, self-monitoring, body image, stress management, barriers and facilitators to weight loss, and others were part of the behavior modification curriculum.

In short, content included exercise, nutrition, and behavior modification components. Exercise topics ranged from the caloric expenditure of some common physical activities to choosing the right apparel to exercise. Exercise behavioral contents involved a motivational setup to increase walking and lifestyle physical activity, in which a pedometer was distributed and planning and log techniques were taught. Nutrition topics dealt, for example, with macronutrient and micronutrient content of the most common foods, energy density, and meal frequency. Behavioral nutrition contents comprised planning for special occasions, using the hunger scale, emotional eating, and preventing lapses, among others.

These contents were expected to have effects on several constructs of the health behavior theories studied in the present investigation. For example the planning techniques should have an effect on intentions, on expected outcomes and in behavioral POC, while the more instructional activities should have interfered with attitudes, perceived barriers and in cognitive POC. In the beginning of each session participants were asked to share with the group their program-related experiences in the previous week. This discussion should have impacted on social support, social norms and self-efficacy, by vicarious learning and also by verbal and social persuasion from both staff and group members. Lastly, the intervention had the underlying goals of improving autonomy and that the participants should choose the tasks that were more enjoyable to them. These are highly motivational factors that have an effect on SDT constructs, accounting specifically for intrinsic motivation.

The sessions were conducted by a team composed by two Ph.D.- and six M.S.-level exercise physiologists, psychologists, or dietitians. Participants were provided with individualized dietary plan and specific physical activity goals, aiming to induce an energy deficit of 300–500 kcal/d, by comparison with baseline values. Participants were informed that weight loss should be understood as a long-term goal, and that 5% weight loss after six months was an appropriate goal.

### Instruments

#### Psychosocial variables

A large battery of psychometric instruments was used in this study and participants were requested to attend two sessions for their completion, in each evaluation period. The instruments were Portuguese validated versions of some of the most used instruments for the constructs under analysis. In this section and throughout the manuscript, variables were divided and are presented in two separate categories: "weight management" and "exercise".

##### Weight management

The SCT weight management-related variables included self-efficacy and outcome expectancy measures. The Weight Efficacy Lifestyle Questionnaire [WEL – [43,44]] is a 20 item instrument from which 5 dimensions and a global sum score can be extracted. For this study only the global score was used (α = 0.95), where higher values represent greater beliefs toward the completion of weight management actions, particularly regarding eating (e.g., "I can resist eating even when others are pressuring me to eat"). Outcome expectancies were derived from the dream weight outcome expectancy score of the Goals and Relative Weights Questionnaire [41,45]. The participants were asked to indicate their dream weight at the end of the program and the difference between this value and the corresponding value at baseline was calculated (these values were presented as a percentage of the baseline weight – for example: if the dream weight was 95 kg and the baseline weight was 100 kg then the dream weight value would be 95%) The aim was to create a score which might reflect a change in expectations for weight loss that was independent of weight change obtained during the program, and also independent of starting weight. For example, an increase in dream weight during the program would reflect a lowering of expectations regarding weight outcomes and a decrease in the importance attributed to achieving that idealized weight value.

The TTM weight management constructs were i) self-efficacy [43,44], ii) the SOC, measured by four questions developed by Suris et al. [32], and iii) the processes of change (POC), assessed by the Weight Processes of Change Scale [WPCS – [32,46]], comprising 40 items that evaluate 10 dimensions (4 items each) divided into behavioral processes (sum of 5 dimensions, α = 0.83) and cognitive processes (sum of 5 dimension, α = 0.90). Higher values of the SOC represent a behavior closer to maintenance and higher values of the POC represent greater cognitive and behavior resources used in the prosecution of weight management.

The TPB weight management constructs were assessed by a set of 18 items [47,48] measuring intentions (4 items; α = 0.93), attitudes (5 items; α = 0.78), subjective norms (3 items; α = 0.71), and PBC (6 items; α = 0.75) towards weight management. Higher values represent greater intentions, attitudes, subjective norms, and PBC.

##### Exercise

Social-cognitive theory exercise-related variables comprise self-efficacy, perceived barriers, and social support. Exercise self-efficacy was measured with the Self-Efficacy for Exercise Behaviors Scale [SEEB – [49,50]], assessing the beliefs that a person can maintain an exercise program for at least six months under varying circumstances. For this study we have used the total score, an average of the 10 items (α = 0.76), where higher scores represent higher self-efficacy. Exercise perceived barriers were assessed with the Exercise Perceived Barriers scale [EPB – [50,51]50, 51]. The average of the 11 items was used as a total score (α = 0.70), where higher values represent greater number and/or degree of perceived barriers to engage in exercise. Social support was measured by the Exercise Social Support [ESS – [52,53]]. The average of the 13 items represents the total score used in this study (α = 0.86). Higher ESS values represent greater social support to participate in exercise.

The TTM exercise related variables were self-efficacy [49,50], SOC, and POC. Exercise stages of change was assessed with six items [27,54], where each item represents a SOC (i.e., pre-contemplation is represented by the value 1 while the maintenance value is 5). POC were assessed by the Exercise Processes of Change [EPC – [54,55]], a 30-item questionnaire that comprises 10 dimensions (3 items each). These dimensions were used to calculate cognitive POC (5 dimensions, α = 0.76), and behavioral POC (5 dimensions, α = 0.75). Higher values represent greater adoption of POC.

The TPB exercise-related variables were assessed through 17 items [27,56] measuring intentions (2 items; α = 0.68), attitudes (7 items; α = 0.72), subjective norms (3 items; α = 0.71), and PBC (5 items; α = 0.80). Higher values represent greater intentions, attitudes, subjective norms and PBC.

We used the Intrinsic Motivation Inventory [IMI – [57,58]], to collect data for the exercise related SDT constructs. The 16 items measure motivation to exercise in the dimensions of interest/enjoyment (α = 0.81), perceived competence (α = 0.68), importance/effort (α = 0.70), and pressure/tension (α = 0.68), each one with 4 items. Because pressure/tension is inversely correlated to intrinsic motivation, this scale was reversed before analysis. A total score can be computed by averaging the 16 items, with higher values representing greater overall exercise intrinsic motivation (α = 0.90).

### Weight

Weight was measured at baseline and four months. A standardized procedure was used where weight was measured twice, to the nearest 0.1 kg (average was used), using an electronic scale (SECA model 770, Hamburg, Germany).

### Data preparation

For correlational analyses, all variables were expressed by the residuals of the 4-month variable value regressed on baseline data. This procedure is recommended by Cohen and Cohen [59] as it creates a value that is orthogonal to the pre-treatment value, representing a more precise measure of change when compared with pre-post subtraction procedures.

### Statistical analysis

The impact of the intervention on weight and psychosocial variables was assessed by paired t-test procedures. Effect size' were calculated, and the criteria to designate its magnitude was the following: < .30 small effect size; .30 to .80 medium effect size; >.80 large effect size [59].

The linear bivariate association between changes in weight and psychosocial variables were assessed by Pearson correlations. Multiple regression models were created to evaluate the multivariate estimates for the associations between psychosocial variables and weight change. The health behavior models' variables were entered separately in seven regression designs (three for the weight management related models and four for the exercise related models). The squared semi-part correlation was calculated to reflect the unique contribution of each predictor to the variance in the outcome variables [59].

## Results

**Figure 1:**
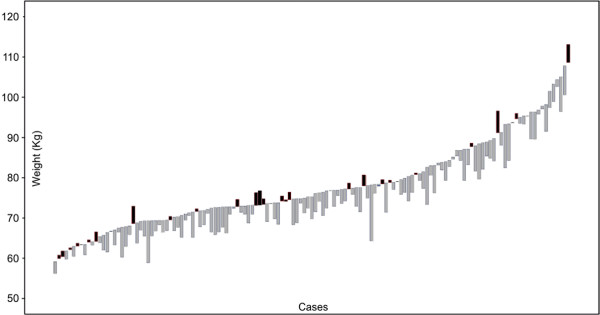
**Weight Change from Initial Weight per Subject**. Each bar represents a participant and their weight change from initial weight (black bars reflect weight gain, grey bars represent weigh loss).

Weight change (WC) showed a large individual variability (-13.85 to 5.38 kg, see Figure 1), with the paired t-test reflecting a significant mean decrease from baseline to four months (-2.94 ± 3.15 kg, t(133)=-10.76, p < .001).

At baseline, about 75% participants reported being at the first three stages of change for exercise, specifically: pre-contemplation (1.5%), contemplation (35.5%) and preparation (37.5%). After the 4-month intervention, these numbers were inverted, as participants were mostly in the action (58.6%) or maintenance (18.0%) stages. Further analysis of the exercise-related psychosocial variables (see Table 1) showed significant changes during the intervention, in the expected direction, with the exceptions of self-efficacy and subjective norms. Behavioral (p < .001, d = 0.85) and cognitive POC (p < .001, d = 0.53), perceived barriers (p < .001, d=-0.38), social support (p < .001, d = 0.48), intentions (p < .01, d = 0.33), exercise interest/enjoyment (p < .001, d = 0.31), perceived competence (p < .001, d = 0.49), importance/effort (p < .001, d = 0.55)and total intrinsic motivation (p < .001, d = 0.50) were the variables that changed the most.

**Table 1:** Descriptive Statistics for Baseline and 4-Month Psychosocial Variables

		Baseline	4 months				
	N	M ± SD	M ± SD	t		95% CI	ES

**Exercise**							
**TTM/SCT**							
Cognitive processes of change	125	48.07 ± 9.15	52.85 ± 8.77	7.49	***	[6.04–3.51]	.53
Behavioral processes of change	125	43.51 ± 10.22	51.94 ± 9.61	9.45	***	[10.19–6.66]	.85
Self-efficacy (ESE)	126	38.38 ± 4.85	37.98 ± 5.59	-.91		[0.46–(-1.23)]	-.08
Perceived barriers (EPB)	126	29.40 ± 6.22	27.04 ± 6.23	-5.26	***	[(-1.47)-(-3.24)]	-.38
Social support (ESS)	127	29.22 ± 6.82	32.79 ± 7.97	5.39	***	[4.88–2.26]	.48
**TPB**							
Intentions	126	12.22 ± 2.04	12.79 ± 1.31	3.04	**	[0.93–0.20]	.33
Attitude	126	42.01 ± 4.28	42.81 ± 3.77	2.31	*	[1.49–0.12]	.19
Subjective norms	126	18.81 ± 2.73	18.54 ± 2.66	-1.04		[0.22-(-0.77)]	-.09
Perceived behavioral control	126	25.55 ± 4.56	26.71 ± 4.58	2.80	**	[1.97–0.34]	.25
**SDT**							
Interest/Enjoyment (IMI)	125	14.79 ± 3.25	15.70 ± 2.68	4.06	***	[1.35–0.47]	.31
Perceived competence (IMI)	125	12.53 ± 2.76	13.81 ± 2.53	7.60	***	[1.62–0.95]	.49
Importance/Effort (IMI)	125	13.42 ± 2.85	14.93 ± 2.60	6.83	***	[1.95–1.07]	.55
Pressure/Tension (IMI)	125	15.12 ± 2.63	15.72 ± 2.55	3.12	**	[0.98–0.22]	.23
Exercise motivation (IMI)	125	55.65 ± 9.21	60.07 ± 8.33	7.41	***	[5.60–3.24]	.50
**Weight Management**							
**TTM/SCT**							
Cognitive processes of change	124	54.81 ± 12.99	57.91 ± 12.50	3.59	***	[4.83–1.39]	.24
Behavioral processes of change	124	50.21 ± 9.46	56.87 ± 10.48	8.39	***	[8.22–5.08]	.67
Self-efficacy (WEL)	125	117.94 ± 31.57	133.61 ± 27.09	6.28	***	[20.61–10.73]	.53
Outcome expectancy (dream weight)	124	60.02 ± 6.31	60.35 ± 5.95	-2.13		[0.02-(-0.65)]	.05
**TPB**							
Intentions	126	25.84 ± 2.69	25.78 ± 2.94	-.23		[0.49-(-0.61)]	-.02
Attitude	126	29.78 ± 4.83	30.83 ± 4.24	2.47	**	[1.88–0.21]	.23
Subjective norms	126	25.04 ± 3.80	24.89 ± 3.45	-.44		[0.50-(-0.79)]	-.04
Perceived behavioral control	126	27.54 ± 4.26	28.51 ± 3.61	2.62	**	[1.70–0.24]	.25

TTM/SCT – Transtheoretical Model and Social Cognitive Theory (as they share the self-efficacy construct they are presented together); TPB – Theory of Planned Behavior; SDT – Self Determination Theory; ESE – Exercise Self-Efficacy; EPB – Exercise Perceived Barriers; ESS – Exercise Social Support; IMI – Intrinsic Motivation Inventory; WEL – Weight Efficacy Lifestyle Questionnaire.

* p < 0.05; ** p < 0.01; *** p < 0.001; ES – Effect Size; 95% CI – 95% Confidence Interval for mean difference

The analysis of the weight management variables showed that the distribution of participants at baseline on the SOC was 50.0% in contemplation, 43.5% in action, and 6.6% in maintenance SOC. Almost all contemplators changed to the action SOC after the intervention (86.7% of the participants), while maintenance was reached by 11.7% of the women. Weight management psychosocial variables did not change as markedly as exercise constructs and different variables emerged as significant, with intentions, subjective norms and outcome expectancies showing no change, while behavioral POC (p < .001, d = 0.67), self-efficacy (p < .001, d = 0.53), cognitive POC (p < .001, d = 0.24), attitude (p < .01, d = 0.23), and PBC (p < .01, d = 0.25) reflected the desired intervention changes.

Pearson correlation was used to analyze associations between predictors and WC (Table 2). The first set of correlation was done between baseline values in predictors and weight change, to explore possible moderator effects. Only weight management SOC, self-efficacy and PBC showed significant results (p < .05). For the correlations with 4-month change in predictors, weight change was associated with most of the putative exercise and weight management variables, most significantly with self-efficacy (both exercise and weight management), and with attitudes and PBC towards weight management (p < .001). All associations were in the expected direction, with self-efficacy, attitudes, and PBC increasing as weight was being lost. Change in importance/effort (p < .01), and POC, social support, intentions, attitude, PBC, and exercise intrinsic motivation (all p < .05), were positively associated with weight loss, while changes in perceived barriers was negatively associated with weight loss (p < .05), as expected.

**Table 2:** Pearson Correlation Between Weight Change and Baseline and 4-Month Change in Psychosocial Scores

	*Baseline*		*4-Month Change*	
**Exercise**				
**TTM/SCT**				
Cognitive processes of change	0.04		-0.18	*
Behavioral processes of change	0.08		-0.18	*
Stages of Change	-0.03		0.11	
Self-efficacy (ESE)	-0.03		-0.29	***
Perceived barriers (EPB)	0.07		0.19	*
Social support (ESS)	0.17		-0.19	*
**TPB**				
Intentions	0.14		-0.19	*
Attitude	-0.14		-0.19	*
Subjective norms	0.04		-0.05	
Perceived behavioral control	0.13		-0.21	*
**SDT**				
Interest/Enjoyment (IMI)	-0.01		-0.11	
Perceived competence (IMI)	0.03		-0.11	
Importance/Effort (IMI)	-0.06		-0.25	**
Pressure/Tension (IMI)	-0.11		-0.02	
Exercise intrinsic motivation (IMI)	-0.05		-0.17	*
**Weight Management**				
**TTM/SCT**				
Cognitive processes of change	0.07		0.01	
Behavioral processes of change	0.14		-0.21	*
Stages of Change	0.22	*	0.04	
Self-efficacy (WMSE)	-0.19	*	-0.42	***
Outcome expectancy	0.02		0.06	
**TPB**				
Intentions	-0.11		-0.17	*
Attitude	-0.12		-0.37	***
Subjective norms	0.03		-0.08	
Perceived behavioral control	-0.18	*	-0.37	***

TTM/SCT – Transtheoretical Model and Social Cognitive Theory (presented together); TPB – Theory of Planned Behavior; SDT – Self Determination Theory; ESE – Exercise Self-Eficcay; EPB – Exercise Perceived Barriers; ESS – Exercise Social Support; IMI – Intrinsic Motivation Inventory; WEL – Weight Efficacy Lifestyle Questionnaire; A negative correlation score indicates a positive association with weight loss; * p < 0.05; ** p < 0.01; *** p < 0.001.

To look further at the predictive power of constructs from behavior change models on WC, we designed a set of multiple regressions, with separate models for the constructs within each theory (Tables 3 and 4). This set was composed by four regression models for exercise TTM, SCT, TPB, and SDT; and three models for weight TTM, SCT and TPB models. All psychosocial scores entered regressions models as independent variables, reflecting 4-month changes (by the use of the baseline residualized 4-month score).

**Table 3:** Multiple Regression Analysis for the Prediction of Weight Change from Weight Management Related Behavior Change Models

**Prediction Variables**	β	sr^2^	p
**SCT – Weight Management**			
Self-efficacy (WEL)	-0.46	20.5%	< 0.001
Outcome expectancy	0.02	0.0%	0.783
	**R**^**2**^**(R**^**2 **^_**adj.**_**)**	20.9% (19.6%)	< 0.001
**TTM – Weight Management**			
Cognitive processes of change	0.16	1.6%	0.108
Behavioral processes of change	-0.23	3.1%	0.027
Stages of Change	0.07	0.5%	0.370
Self-efficacy (WEL)	-0.45	19.4%	< 0.001
	**R**^**2**^**(R**^**2 **^_**adj.**_**)**	26.8% (24.3%)	< 0.001
**TPB – Weight Management**			
Intentions	0.00	0.0%	0.963
Attitude	-0.24	4.0%	0.017
Subjective norms	0.01	0.0%	0.892
Perceived behavioral control	-0.24	3.7%	0.022
	**R**^**2**^**(R**^**2 **^_**adj.**_**)**	17.6% (14.8%)	< 0.001

All variables were entered in the model; theory-related variables were entered separately in three regression models; TTM- Transtheoretical Model; SCT Social Cognitive Theory; TPB – Theory of Planned Behavior; WEL – Weight Efficacy Lifestyle Questionnaire; R^2 ^_adj._, R square adjusted; sr^2^, semi-partial correlation coefficient.

**Table 4:** Multiple Regression Analysis for Weight Change from Exercise Related Behavior Change Models

**Prediction Variables**	β	sr^2^	p
**SCT – Exercise**			
Self-efficacy (ESE)	-0.23	4.6%	0.013
Perceived barriers (EPB)	0.10	0.8%	0.296
Social support (ESS)	-0.14	1.8%	0.119
	**R**^**2**^**(R**^**2 **^_**adj.**_**)**	11.4% (9.2%)	0.002
**TTM – Exercise**			
Cognitive processes of change	-0.08	0.4%	0.481
Behavioral processes of change	0.01	0.0%	0.931
Stages of Change	0.05	0.2%	0.619
Self-efficacy (ESE)	-0.26	5.2%	0.010
	**R**^**2**^**(R**^**2 **^_**adj.**_**)**	9.4% (6.3%)	0.019
**TPB – Exercise**			
Intentions	-0.08	0.4%	0.491
Attitude	-0.10	0.7%	0.344
Subjective norms	0.03	0.1%	0.733
Perceived behavioral control	-0.12	1.0%	0.258
	**R**^**2**^**(R**^**2 **^_**adj.**_**)**	5.9% (2.8%)	0.116
**SDT – Exercise**			
Interest/Enjoyment (IMI)	-0.04	0.1%	0.735
Perceived competence (IMI)	0.04	0.1%	0.759
Importance/Effort (IMI)	-0.26	4.8%	0.015
Pressure/Tension (IMI)	0.05	0.2%	0.630
	**R**^**2**^**(R**^**2 **^_**adj.**_**)**	6.4% (3.3%)	0.091

All variables were entered in the model; theory-related variables were entered separately in four regression models; TTM- Transtheoretical Model; SCT Social Cognitive Theory; TPB – Theory of Planned Behavior; SDT – Self Determination Theory; ESE – Exercise Self-Eficcay; EPB – Exercise Perceived Barriers; ESS – Exercise Social Support; IMI – Intrinsic Motivation Inventory; R^2 ^_adj._, R square adjusted; sr^2^, semi-partial correlation coefficient

Weight management variables presented the stronger models, particularly the TTM (R^2 ^= 26.8%, p < .001), mostly due to changes in self-efficacy, which independently explained 19.4% of WC variance, seconded by behavioral POC with 3.1%. The SCT represented the next strongest model (R^2 ^= 20.9%, p < .001), with changes in self-efficacy alone contributing 20.5% to the explained variance. The model for TPB explained 17.6% (p < .001) of the variance in weight change, with attitude and PBC showing similar semi-part correlation values (≈4%, p < .05). All other weight management psychosocial constructs did not contribute significantly to the models.

Table 4 shows the results of the four regression models using exercise-related variables as independent variables. As could be anticipated by the bivariate analysis, predictive power was generally lower for these models in comparison with weight management analyses. The SCT was the strongest model (R^2 ^change = 11.4%, p = .002), seconded by TTM (R^2 ^change = 9.4%, p = .019). Change in self-efficacy was the only variable that significantly added predictive power to these models (4.6% and 5.2%, respectively). The other models did not account significantly to weight change, although the importance/effort dimension in the SDT model independently contributed with 4.8% of the explained variance (p = .015).

## Discussion

This study was conceived to analyze how changes in key psychosocial exercise- and weight management-related variables, derived from four important health behavior theories, would predict weight change during a behavioral obesity treatment short-term intervention. Weight change was significantly predicted by several single variables and by health behavior change theories/models as a whole. The following were this study's primary findings: a) Change in eating/weight management self-efficacy was the single best correlate of weight reduction, though several other variables were also associated with weight outcomes (e.g., change in PBC and attitudes regarding weight management and exercise, increases in the importance attributed to exercise, and change in some self-report behavioral processes of change) ; b) About 20–30% of the variance in weight change was explained by the best prediction models and most showed statistically significant prediction (i.e., R^2^) scores; c) Theories that included self-efficacy (TTM and SCT) presented the stronger regression models, and d) Change in variables and models related to weight management had higher predictive power than those from exercise-related models.

Not many studies have used a mediating variable model framework to verify how weight change during obesity treatment programs is predicted by change in psychosocial variables included in health behavior change theories [10]. Even fewer studies have directly compared several constructs from health behavior change theories within the same sample and intervention. The current study's design was mindful of these shortcomings, as it used an extensive battery of measures, covering some of the most cited constructs in paradigmatic health behavior change theories, and analyzed not only baseline but also post-intervention values, i.e., changes that occurred during the weight management program.

Change in variables from the health behavior models under analysis was generally predictive of weight outcomes. We analyzed change independently of baseline values, so these changes most likely occurred as an effect of the intervention and may be considered as potential mediators of the intervention outcomes [60]. This potential mediator effect should be analyzed with a control group design in future studies. Even though the present study analyzed short-term outcomes, the overall predictive power was in line with what has been found in similar previous studies, reaching 20–30% of explained variance in weight change [10]. The stronger predictive power in the weight management models, where items in the respective instruments were often addressing eating-related aspects (most especially the self-efficacy measure – the WEL) was an expected result since this was a short-term intervention. The more immediate effects on weight loss from dietary changes is well documented, whereas exercise behavior has more frequently been associated with long-term weight loss [61,62]. Also, even when questionnaire items were phrased regarding weight management in general, it is likely that they were interpreted by participants as being highly related to eating behaviors and dieting; the general perception, at least in Europe, still remains that to lose weight successfully one needs to diet, more than to adopt any other behavioral change [63].

The stronger models, TTM and SCT, were weight management-related and both included self-efficacy. We found few studies that have analyzed *change *in self-efficacy as a predictor, but they have generally confirmed the present findings (i.e., greater improvements leading to greater weight losses) [19,64]. In the present study, we used a slightly different change variables than in previous research (i.e., residuals as opposed to pre-post subtraction), but found similar results, indicating that self-efficacy improvement predicts weight change independently of its baseline scores. The consistency of these results can be explained by self-efficacy theory itself, since efficacy beliefs are presented as a function of enactive mastery experiences, vicarious learning, verbal persuasion, and physiological and emotional activation [8]. It could be hypothesized that, as participants were losing weight, they improved their self-efficacy towards weight loss behaviors, by means of enhanced mastery experiences and possibly positive emotional activation from being able to getting closer to their goals. Another factor that could have contributed to the changes in self-efficacy was verbal persuasion by the intervention team and peers. Jeffery [39] reviewed the role of theory-based interventions conducted within his work and pointed out self-efficacy as the most important predictor of weight outcomes. US obesity treatment guidelines also reflect the importance of considering self-efficacy on weight loss treatment [65,66]. Interestingly, baseline self-efficacy values have shown mixed evidence as prospective predictors of weight loss [5], raising the question of reciprocity between self-efficacy and outcomes; heightened self-efficacy values can be a reflection of weight loss results as much as a predictor of weight loss. This question remains unresolved by the present results.

Exercise processes seemed to be substantially influenced by the intervention, which included information on how to cope with common barriers, recommended exercises (walking was strongly reinforced by means of a pedometer and self-monitoring), scheduling techniques, physiologic and psychological benefits of exercise, and how to use/find available resources. Nevertheless, exercise-related variables and models were only moderately associated with weight outcomes with self-efficacy again showing the highest bivariate and multivariate associations. It is interesting to note that despite its association with weight loss, mean exercise self-efficacy scores did not change significantly during the intervention. As pointed out before [5], it is possible that at the initial stages of behavior engagement, some of the cognitive evaluations could be overstated by the lack of knowledge of "what it takes" to comply with regular action toward that behavior. At baseline, most of our participants were sedentary and exercise contemplators, so they could be overestimating their abilities to engage in exercise. This is similar to what was found in a previous study [32], where new SOC for weight management were proposed to adjust for that reality. Analogous explanations were advanced by Martin et al. [64].

Self-Determination Theory was only evaluated regarding exercise constructs and represented a stronger model than the TPB, with the importance/effort dimension emerging as a single predictor from SDT. The intervention sessions repeatedly reinforced the importance of exercise for the success in weight management, especially for long-term outcomes, for instance by citing results from the National Weight Control Registry results [67]. As a consequence, this should have led, at least at a cognitive level, to an increase on the positive evaluations and perceived importance of exercise. Recently, Teixeira et al., [35] showed that early changes in exercise intrinsic motivation variables predicted long-term weight change, beyond and above short-term weight variation and eating-related variables. In two previous studies analyzing baseline predictors of weight loss, we have also shown that another SDT-related construct, self-motivation [68], was predictive of weight change [21,41]. These and other results [36,69] indicate that SDT could play an important explanatory role in long-term weight control, where exercise is believed to exert most of its positive impact [62,70].

Limitations of this investigation include self-reported data, the absence of a control group, a relatively small sample, and the lack of complete evaluation for some models (e.g. social support, perceived barriers, and SDT constructs for weight management), mostly due to the absence of validated Portuguese questionnaires for these constructs. Also, some of the constructs were analyzed with less than ideal measures, such as outcome expectancies. Finally, multiple measures collected during the 4-month program, instead of only pre and post results, would have improved our assessment of the psychosocial variables by better describing changes in each construct throughout the program.

## Conclusion

In sum, we observed that theories that comprise self-efficacy are the most predictive of weight change and that weight management- and eating-related constructs and theories better explain the variance in short-term outcomes, compared to exercise models. To a lesser extent, exercise theories were also predictive. However, their predictive power is expected to increase in longer-term analyses, especially for variables related to intrinsic motivation and SDT. This is in line with recent results in a very similar intervention, where psychosocial eating variables better predicted 4-months results while exercise motivation constructs were superior correlates of 16-month weight loss [35]. In the future, researchers should also look at other theories and variables that could help explain weight outcomes. For example, variables related to affect and subjective well-being [71] as well as body image constructs [72] could offer important insight on how people make decisions about weight control tasks. Because these variables, i.e., body image and subjective well-being, should be enhanced by exercise adherence, these studies would also improve our understanding of the relationship between exercise behaviors and successful weight control, beyond their direct effect on caloric expenditure.

## Competing interests

The author(s) declare that they have no competing interests.

## Authors' contributions

ALP and PJT conceived the study and drafted the manuscript. ALP performed the statistical analysis, was responsible for psychometric assessments and participated in the study's implementation. TLB, SSM, CSM, and JTB actively participated in the study implementation and in data collection. SOS participated in the study design and in the selection of psychosocial predictors. LBS is a principal investigator in the research trial. All authors read and approved the final manuscript.
